# Improving Wellness of Operating Room Personnel: A Light-Based Intervention on Perceived Nursing-Related Stress

**DOI:** 10.3389/fpsyt.2021.718194

**Published:** 2021-09-07

**Authors:** Gilles Guerrier, Dimitri Margetis, Christine Agostini, Zakia Machroub, Sophie Di Maria

**Affiliations:** ^1^Department of Anaesthesia and Intensive Care, Hôpital Cochin, Assistance Publique-Hôpitaux de Paris, Paris, France; ^2^Department of Anaesthesia and Intensive Care, Hôpital de la Pitié-Salpêtrière, Assistance Publique-Hôpitaux de Paris, Paris, France

**Keywords:** well-being, operating room, light based device, nursing staff, crossover study

## Abstract

**Background:** Nursing is an emotionally demanding and physically draining occupation. Well-being of health care workers is essential to achieve success in care and have good cooperation relationships with other health professionals.

**Objective:** The purpose of this study was to evaluate the effectiveness of a light-based intervention on perceived nursing-related stress in health care personnel working in an operating room environment.

**Methods:** A total of 84 nurses participated in this randomized, cross-over controlled study. Intervention consisted of 4 weeks of bright blue-enriched light exposure using a LED head-mounted portable device (*n* = 42) or no light exposure (*n* = 42) separated by a 2-week washout period in a crossover fashion. Participants completes questionnaires for the Nursing Stress Scale (NSS).

**Results:** Intervention and control groups were comparable in terms of demographics, with a median age of 34 (IQR: 27–49) and 69 (82%) female. The mean baseline NSS score was similar in both groups before intervention. The NSS score of the intervention group was significantly lower after intervention than the baseline score: the NSS score difference before and after intervention was 15.1 (SD 7.6) (*p* < 0.001) and 19.7 (SD 7.5) (*p* < 0.001) during the two successive periods of intervention, respectively. The cross-group comparison after intervention showed a significantly higher NSS score difference after intervention in the intervention group than the control group: 15.1 (SD 7.6) vs. 1.4 (SD 8.4) (*p* < 0.001) and 19.7 (SD 7.5) vs. 1.7 (SD 8.9) (*p* < 0.001) during the two successive periods of intervention, respectively.

**Conclusion:** Alternative person-directed initiatives should be considered to improve the well-being of the health workforce in operating rooms, especially during the coronavirus pandemic.

## Introduction

Well-being of health care workers is essential to achieve success in care and have good cooperation relationships with other health professionals, including physicians (1). Nursing is an emotionally demanding and physically draining occupation, leading to increased incidence of sick-leave and burnout rates. Field experience shows that in-person interventions to address the high levels of stress among non-medical health care workers is challenging to be implemented in hospitals. In addition, research on the interventions for their effectiveness on nursing staff's well-being at work is scarce. Bright light therapy (BLT) is receiving growing interest as a potential efficient means to treat an increasing number of disorders or disabilities, including, seasonal (1) and non-seasonal major depression (2), sleep and circadian rhythm disorders (3), and addiction (4). The purpose of this study was to evaluate the effectiveness of a light-based intervention on perceived nursing-related stress in an operating room environment. We hypothetized such person-directed initiatives may improve the well-being of the health workforce.

## Materials and Methods

With approval from the Ethics Committee of the French Society of Ophthalmology (IRB 00009254 Société Française d'Ophtalmologie), a cross-over randomized controlled trial was conducted in two teaching hospitals in Paris, France. All the scrub and circulating working in the ambulatory surgery ward was offered to participate to the study. All participants gave their written informed consent. They were randomly assigned to BLT or no light group using a web-based randomization tool in a crossover design. In the first group, participants initially received 4 weeks of active BLT (period A) and then 4 weeks of no light (period B). In the second group, participants initially received 4 weeks of no light (period B) and then 4 weeks of active BLT (period A). The treatment periods were separated by a washout period of 2 weeks. Each of the subjects were therefore assigned to one of the two possible intervention sequences (A then B, group AB or B then A, group BA, separated by a washout period). Participants in the no-light group did not wear any portable device. Patients were asked to answer evaluation questionnaires before and after each treatment period.

Active BLT was delivered through a LED head-mounted portable device that includes a holographic surface reflecting light toward the lower retina such that sight is not hindered (Luminettes®, Lucimed, Villers-Le-Bouillet, Belgium). Emitted light was blue enriched light with a maximum wavelength of 468 nm. Participants were instructed to wear the device for 45 min every morning at home at the same time daily for 1 month. Patients were allowed to read or to perform simple daily tasks while wearing the device. A calendar was provided for participants to record their daily use of the device. The assessors were not aware of the randomization process.

The outcome measures were perceived nursing-related stress evaluated by the Nursing Stress Scale (NSS) (5) covering a total of 34 items, including questions concerning nursing professionals and work, problems about time allocation and workload, problems regarding work environment and equipment, problems associated with patient care, and problems related to management and interpersonal relationships. A four-point rating is used for each item. A higher score indicates a higher degree of stress.

The scale was divided into seven factors as follows:

- Workload included breakdown of computer, perception of too many non-nursing tasks, and time pressures regarding the provision of nursing care and emotional support.
- Death and dying explored the perceptions of participants about the frequency to which they difficulty when dealing with very ill and dying patients.
- Inadequate preparation explored the frequency of which participants felt inadequately prepared for their role in dealing with difficult questions and in the provision of emotional care to both the patient and relatives.
- Lack of staff support looked at participants' view on the support available to them in the clinical setting.
- Uncertainty concerning treatment focused on the frequency in which participants felt that there was inadequate information for patients regarding treatment, inappropriate treatment and uncertainty regarding the working of medical equipment.
- Conflict with physicians focused on the frequency of physician conflict and conflict regarding appropriate medical treatment.
- Conflict with other nurses was concerned with the amount of times that participants felt that they had disagreement with the nursing supervisor and difficulty working with particular nurses within and beyond the ward.

Each item is scored according to the frequency with which these situations are assessed as stressful, from (0) never, (1) sometimes, (2) frequently to, (3) very frequently. The greater frequency of work stressors experienced by the participant is indicated with a higher score. Side effects were also monitored.

The test–retest reliability of the NSS was 0.81 with satisfactory internal consistency (Spearman–Brown coefficient = 0.79; Guttman split-half coefficient = 0.79). This tool has demonstrated validity in the measurement of stress (6, 7).

### Statistical Analysis

Quantitative data were analyzed with descriptive statistics comparing mean scores for each group and focusing on the individual factors in the NSS using a computer software package SPSS version 12 (SPSS Inc., Chicago, IL, USA). Data are expressed as mean and SD unless otherwise indicated. Paired analysis was performed stratified by the order of exposure: *t*-tests were used to compare parameters between groups and Pearson's Chi Square tests were used to compare categorical variables. Patients were considered as dependent variables and treatments (BLT and placebo) and pre- and post-treatment evaluations as independent variables. Bonferroni *t*-test was used to assess the all pairwise multiple comparison procedures. The level of significance was set at *p* < 0.05.

### Endpoints

The primary endpoint of this study was the change in scores for NSS before and after active BLT compared with no intervention.

## Results

Between November 2018 and January 2019, there were 45 respondents in the intervention and control groups, respectively (participation rate 68%). After the intervention, due to the loss of some data of three respondents in the control group and three respondents in the intervention group, only 42 respondents in the control group and 42 respondents in the intervention group were actually included in the analysis. No significant differences were shown in mean age, sex ratio, marital status, highest level of nursing education, and baseline NSS scores of the two groups. The 84 participants had a median age of 34 (IQR: 27–49), and 69 (82%) of them were female. They were single, married, living with partner and divorced/separated in 27% (*n* = 23), 23% (*n* = 19), 40% (*n* = 34), and 8% (*n* = 7), respectively (Table 1). Four participants reported slight headache when wearing glasses but did not stop using BLT when needed. The mean baseline NSS score was similar in both groups before intervention during the AB period: 83.5 (SD 8.1)] and 84.3 (SD 8.1) in the control group and the intervention group, respectively (*p* = 0.7). No significant difference was seen in the mean NSS scores after intervention in control groups: the NSS score difference before and after intervention was 1.4 (SD 8.4) (*p* = 0.16) and 1.7 (SD 8.9) (*p* = 0.12) during periods AB and BA, respectively. The NSS score of the intervention group was significantly lower after intervention than the baseline score: the NSS score difference before and after intervention was 15.1 (SD 7.6) (*p* < 0.001) and 19.7 (SD 7.5) (*p* < 0.001) during periods AB and BA, respectively. The cross-group comparison after intervention showed a significantly higher NSS score difference after intervention in the intervention group than the control group: 15.1 (SD 7.6) vs. 1.4 (SD 8.4) (*p* < 0.001) and 19.7 (SD 7.5) vs. 1.7 (SD 8.9) (*p* < 0.001) during periods AB and BA, respectively. During the BA period, the mean baseline NSS score was higher in the intervention group compared to the control group: 70.8 (SD 9.0)] and 80.9 (SD 8.3), respectively (*p* = 0.04) (Table 2).

**Table 1:** Participants characteristics.

	**Groupe 1** ***n* = 42**	**Groupe 2** ***n* = 42**
**Gender** ***n*** **(%)**		
Male	7 (17)	8 (19)
Female	35 (83)	34 (81)
**Age [IQR]**	33 [26–48]	35 [23–51]
**Marital status** ***n*** **(%)**		
Single	11 (26)	12 (28)
Married	10 (24)	9 (21)
Divorced/Separated	5 (12)	2 (5)
Living with partner	15 (36)	19 (45)
Widoweda	1 (2)	0
**Highest level of nursing education** ***n*** **(%)**		
Bachelor's in nursing	33 (79)	37 (88)
Master of Science in nursing	8 (19)	5 (12)
Ph.D.	1 (2)	0
**Clinical nurse manager** ***n*** **(%)**	4 (9)	3 (7)

**Table 2:** Comparison of the two groups in Nursing Stress Scale after the intervention.

	**Before intervention NSS Mean (SD)**	**After intervention NSS Mean (SD)**	**NSS difference before and after intervention NSS Mean (SD)**	***p* of intra-group comparison**	***p* of cross-group comparison of difference**
**First period (AB)**					
Control group (*n* = 42)	83.5 (8.1)	82.1 (7.4)	1.4 (8.4)	0.16	<0.001
Intervention group (*n* = 42)	84.3 (8.3)	64.2 (9.4)	15.1 (7.6)	<0.001	
**Second period after washout (BA)**					
Control group (*n* = 42)	70.8 (9.0)	72.5 (12.9)	1.7 (8.9)	0.12	<0.001
Intervention group (*n* = 42)	80.9 (8.3)	61.2 (9.1)	19.7 (7.5)	<0.001	

## Discussion

The main finding of our study is a significant positive impact of BLT on well-being among the nurses working in operating rooms. Our result is consistent with other reports studying the effect of luminotherapy in health car staff working under artificial light condition (8). This finding is particularly interesting since this intervention is non-invasive and well-accepted to improve the mental state of nursing staff. Previous reports have explored other alternative therapies such as mindfulness-based stress reduction (9) but to our knowledge, this is the first published study using BLT to reduce occupational stress and stress response of operating room nurses. The reason why BLT can reduce work stress is related to its effect on biological clock synchronization with the environmental day-night rhythm and to shift the circadian rhythm (10, 11). Evidence suggests that the effects of BLT on mood disorders are linked to enhanced sleep and rhythms of melatonin and cortisol (12). The synchronization of the biological clock by BLT involves the hypothalamus-pituitary-adrenal gland axis regulating the secretion of cortisol in response to stress (13).

In our study, nurses suffer from high levels of stress at work. This finding is consistent with the literature suggesting that stress at work of operating room nurses is high (14, 15). The operating room is a complex, high-risk environment, with a high potential for adverse events. The specificity of the work of operating room nurses determines greater work stress, which reduces the level of mental health of the nursing staff. In addition, concerns are emerging around psychological conditions of heath care workers due to workload and task shifting induced by the COVID-19 pandemic (16), and ever-growing pressures upon healthcare service budgets. Signs of mental health problems among health care workers during disease outbreaks have been observed in many healthcare settings (17), both among doctors and nurses (18). Improving well-being at work may be cost-effective by raising efficiency and motivation in daily tasks. The mental stress experienced by operating room nurses negatively affects surgical performance, including decision making and communication (19–21). It is therefore important to implement interventions aiming at mitigating the effects of stressors on both technical and non-technical skills of the surgeon and to lower the level of mental stress of the operating room team.

Several limitations should be mentioned. First, compliance rate could not be checked. However, portable individualized light therapy is associated with high compliance, since one can partly continue one's daily activities during light administration. Second, the limited participation rate makes it difficult to generalize the effectiveness of the intervention beyond our population. The reasons for refusal and exploring ways in which participation rate could have been optimized deserve further consideration. Third, the study subjects were mostly female, so extrapolation to male nurses is not warranted without demonstrating this. Fourth, the short washout period may have confound the result in a cross over design. However another report suggested that light therapy effects are short-lasting (22). A final limitation is the lack of a placebo arm, which always represents a significant problem in light treatment trials since there are multiple potential confounders that could have influenced the sense of well-being. However, the crossover design could be an advantage for examining symptoms of ill being at work which may be influenced by confounding factors such as medication, lifestyle, and co-morbidities.

## Conclusion

A brief course of treatment with morning bright light was well-tolerated by nursing staff and improved their well-being at work. These encouraging results suggest considerable potential for light therapy. Such person-directed initiative may be particularly useful to optimize the mental health of nursing staff in this persistent COVID-19 pandemic context.

## Data Availability Statement

The raw data supporting the conclusions of this article will be made available by the authors, without undue reservation.

## Ethics Statement

The studies involving human participants were reviewed and approved by Ethics Committee of the French Society of Ophthalmology (IRB 00009254 Société Française d'Ophtalmologie). The patients/participants provided their written informed consent to participate in this study.

## Author Contributions

GG and SD: study design and writing of first draft. GG, DM, CA, ZM, and SD: participants' recruitment and data collection. GG: data analysis. All authors critically revised the article, approved the final version to be published, and are accountable for all aspects of the work.

## Conflict of Interest

The authors declare that the research was conducted in the absence of any commercial or financial relationships that could be construed as a potential conflict of interest.

## Publisher's Note

All claims expressed in this article are solely those of the authors and do not necessarily represent those of their affiliated organizations, or those of the publisher, the editors and the reviewers. Any product that may be evaluated in this article, or claim that may be made by its manufacturer, is not guaranteed or endorsed by the publisher.
